# Endometrial mesenchymal stem stromal cells in mature and immature sheep: An in vitro study

**Published:** 2018-02

**Authors:** Farnaz Ghobadi, Farhad Rahmanifar, Davood Mehrabani, Amin Tamadon, Mehdi Dianatpour, Shahrokh Zare, Iman Razeghian Jahromi

**Affiliations:** 1 *Stem Cells Technology Research Center, Shiraz University of Medical Sciences, Shiraz, Iran.*; 2 *Department of Basic Sciences, School of Veterinary Medicine, Shiraz University, Shiraz, Iran.*; 3 *Department of Human Genetics, School of Medicine, Shiraz University of Medical Sciences, Shiraz, Iran.*; 4 *Cardiovascular Research Center, Shiraz University of Medical Sciences, Shiraz, Iran.*; * *Davood* Mehrabani and Amin Tamadon are co-corresponding authors.

**Keywords:** Endometrium, Mesenchymal stromal stem cells, Characterization, Differentiation, Sheep

## Abstract

**Background:**

Endometrial mesenchymal stem stromal cells (EnMSCs) are critical for uterine function, repair, and regeneration.

**Objective:**

This study introduced isolation technique of EnMSCs and compared the characteristics of EnMSCs in mature and immature ewes.

**Materials and Methods:**

Endometrial tissue samples from the uterus of 10 ewes were collected from the slaughterhouse. Endometrial cells were isolated from tissue using cold incubation and then chopping and treating was performed with collagenase type I. Isolated cells were cultured in cell culture medium and then attached cells to flasks were harvested as EnMSCs and subcultured. To enumerate the cells, the population doubling time (PDT) was determined and 2.2×10^4^ cells in passage 4 were seeded into 24-well culture plates to compare the growth curves of isolated cells. Reverse transcription polymerase chain reaction (RT-PCR) was performed for detection of CD34 and CD73 markers. The osteogenic and adipogenic potential of isolated cells were determined using differentiation tests.

**Results:**

EnMSCs adhered to the flasks and displayed spindle-shape. Based on findings of the cell count and the growth curves, the EnMSCs growth was significantly more prominent in immature ewes in comparison to mature sheep. The PDT of EnMSCs in immature ewes was about 21 hr whereas this time period was two times higher (45 hr) in mature sheep. RT-PCR analyses of EnMSCs were positive for CD73 and negative for CD34. EnMSCs were differentiated into osteoblasts and adipocytes**.**

**Conclusion:**

Based on mesenchymal stem cells characters confirmed in EnMSCs, they can be a candidate for cell therapy and regenerative medicine.

## Introduction

The uterus is one of the essential organs of reproduction in most mammalian species and has one of the most active tissues in terms of tissue remodeling. According to the histological divisions, the endometrium is the inner layer of the uterus and is divided physiologically into 2 layers; the functionalis (nearest to lumen) and the beneath basalis layer (1). Mesenchymal stem stromal cells (MSCs) have been determined in humans and animals such as sheep, cow, and mouse (2-7). They are critical for cyclic renewal and uterine function and contribute to repair and regeneration of normal endometrium (8, 9). 

A prominent regenerative capacity of endometrium in human due to the highly proliferative character of endometrial MSCs (EnMSCs) and also immuno-privilege behavior of EnMSCs, introduce them a new source of cell therapies and candidate them as easily accessible cells for regenerative medicine (10-14). 

For stem cell therapy of premature ovarian failure and transplantation in chronic diseases such as diabetes and Parkinson's disease, EnMSCs can be ideal cells (15, 16). Anti-inflammatory effects of EnMSCs were confirmed promoting wound repair with new tissue growth and minimal fibrosis (13). Furthermore, differentiation of EnMSCs into ectodermal and mesodermal cellular lineages, such as neural cells, hepatocyte, osteoblasts and heart muscle cells have been reported (17-24).

EnMSCs is critical for future cell based therapies, regenerative medicine and artificial organ reconstitution for a female with the endometrial disorder. Introducing an animal model for the study of human EnMSCs can provide an available cell source for above mentioned purposes (sheep uterus is easily available in slaughterhouses). However, EnMSCs are recently isolated and characterized in ewe (5), but there was no data available in literature to compare the growth kinetic and population doubling time (PDT) of EnMSCs between mature and immature ewes. 

This study was designed to introduce a new chilling method for EnMSCs isolation and determine whether the uterine endometrium of mature and immature ovine contained populations of EnMSCs.

## Materials and methods

**Uterine sampling**

The uterus is a part of an animal carcass which removes from meat processing in industrial slaughterhouse according to Islamic rules and has been used for compost production. Therefore, uterine tissues of 10 healthy mature (approximately 4 to 5 yr-old) and immature (approximately 1 yr-old immature) Fars native ewes (n=5) were collected from Shiraz Industrial Slaughterhouse located in Shiraz, Iran. In cold plastic bags (1^o^C), ovine uteruses were transferred to Stem Cell Laboratory of Stem Cells Technology Research Center. Puberty was determined according to the ovarian condition and the symmetry of uterine horns. Immaturity of ewes was confirmed if small follicles were present and corpus luteum on the surface of ovaries was absent, together with small symmetrical horns in the uterus. Whereas, the existence of corpus luteum on the ovary was regarded as the sign of ovulation and with relatively asymmetrical large uteri horns that were the evidence of previous gestations and maturity of ewes.

**EnMSCs isolation and culture**

In the laboratory, the external surface of the uterine tissue was rinsed well with warm sterile normal saline. Under laminar flow hood condition (Class II, Jal Tajhiz, Iran) using sterile surgical instruments, three full-thickness sections were randomly done on three parts of the uterine tissue (two on both horns and one on the uterine body) to provide the needed endometrial samples. To separate endometrium from myometrium, using a forceps and keeping the surrounding area and then pulling up the internal layer which was exposed out, these two layers were easily isolated by gentle pulling. Then, the three endometrial samples (each 10×10×5 mm^3^) including cotyledon and inter-cotyledonary spaces were subsequently removed. The uterine samples were separately suspended in 15 ml Falcon tubes containing Dulbecco`s modified eagle’s medium (DMEM, BioWest, France) supplemented with 10% fetal bovine serum (FBS, BioIdea, Iran) and 1% penicillin/ streptomycin (BioWest, France).

After cold incubation of the uterine samples at 4^o^C for 24 hr, chopping process was performed in a sterile Petri dish with a sterile scalpel blade. In order to digest the intercellular junctions between cells of the endometrial surface, the chopped uterine tissues were treated with collagenase type I (Invitrogen, USA) in 37^o^C for 45 min in a CO_2_ incubator (Memert, Germany). In order to neutralize the effect of an enzyme, an equal volume of DMEM/10% FBS media was added. The resultant was a solution containing many types of cells (including the EnMSCs), enzyme and the media. The Falcons were then centrifuged at 1200 rpm for 5 min and after removal of the supernatant, the cell pellets were kept for further follow-up. The cell pellets were suspended by fresh DMEM/10% FBS media and cultured in T25 flasks were incubated at 37^o^C, 5% CO_2_ and saturated humidity.

After 12 days of incubation (P0=primary culture), the cells were exposed to trypsin (Sigma, USA), incubated for 5 min and after enzyme neutralization with DMEM/10% FBS, centrifuged sediment cells were suspended in medium and transferred into three T25 flasks (P1=the first passage). After 3 days of incubation, the flasks were brought out from the incubator and using an inverted light microscope (Nikon, Japan) they were observed. After 5 to 6 days, when the adherent spindle fibroblast-like cells were 80% confluent, they were subcultured. To determine the number of live and dead cells, the cells were stained using trypan blue (Sigma, USA) in each passage by a Neubauer chamber.

**Freezing and thawing**

After counting the cells in each passage, half part of the cells was transferred into the next passage and the other was frozen. To freeze the cells, 50% DMEM, 40% FBS and 10% dimethyl sulphoxide (DMSO, MP Bio, USA) was used as the freezing medium. Because of the cell toxicity nature of DMSO substance in room temperature, the cells suspended in freezing medium were transferred to a freezer (-24^o^C) immediately for 1 hr and then to another freezer (-70^o^C) for 24 hr and finally for long-term storage into liquid nitrogen (Atocel, Austria). To evaluate their survival after thawing, 10 days after freezing, the cryotubes were removed from the liquid nitrogen. Tubes were rapidly warmed in a 37^o^C water bath and the same volume of DMEM/10% FBS culture medium was immediately mixed with cell suspensions in a Falcon tube. The tube was centrifuged at 1200 rpm for 7 min. Cell pellet was suspended in a fresh DMEM/10% FBS culture medium. Cell viability was evaluated by the trypan blue exclusion test (0.4% trypan blue in phosphate buffered saline, PBS) and calculated according to the formula: 

$\mathrm{Cell viability}=\frac{\mathrm{Number of viable cells}}{\mathrm{Total number of cells}}\times 100$

**Growth curve analysis**

Experiment 1: To define the appropriate number of cells for seeding and also the optimum surface area for culturing of the immature ewes EnMSCs, the cells at passage 4 were seeded into two 12-well plates at a density of 2.2×10^4^ cells per well, two 24-well plates at a density of 2.2×10^4^ cells per well and two 24-well plates at a density of approximately 1.7×10^4^ cells per well and all plates were transferred into CO_2_ incubator at 37^o^C, 5% CO_2_ and saturated humidity and followed for eight days. The number of cells was counted daily (Three wells per plate in each time per group).

Experiment 2: After achieving the best growth rate with the appropriate surface and initial cell number for seeding EnMSCs of an immature ewes, to compare the growth of EnMSCs between the mature and immature ewes in the same conditions, the cells in passage 4 were cultured in 24-well plates at a density of approximately 2.2×10^4^ cells per well. The cells were incubated at 37^o^C, 5% CO_2_ and saturated humidity for 8 days. The number of cells was counted daily (Three wells per plate in each time per group).

Using GraphPad Prism software (San Diego, CA, USA), the mean number of cells at each counting time was plotted. The PDT was calculated using the formula (7):

$\mathrm{PDT}=T\times \frac{\mathrm{ln2}}{\ln(\frac{\mathrm{Xe}}{\mathrm{Xb}})}$

Where T is the number of days for incubation, ln is natural logarithm, Xe or Xb are the cell numbers determined at the end or beginning of the incubation time, respectively.

**Characterization by reverse transcription polymerase chain reaction (RT-PCR)**

By the RT-PCR method, the expressions of markers of MSCs and hematopoietic stem cells (HSCs) were determined. Briefly, the total RNA was extracted by the column RNA isolation kit (Denazist-Asia, Iran). Then total RNA concentration was evaluated by spectrophotometry. In next step, the complementary DNA (cDNA) was synthetized from the RNA samples using AccuPower Cycle Script RT PreMix Kit (Bioneer, Korea). Briefly, 0.5 µg of total RNA was used for each reaction to reach a volume of 20 µL with the diethyl pyrocarbonate water. Then, 12 thermal cycles were undertaken; primer annealing for 30 sec at 20^o^C, cDNA synthesis for 4 min at 42^o^C, and melting secondary structure and cDNA synthesis for 30 sec at 55^o^C. After the final step of the cycling, inactivation was done for 5 min at 95^o^C. In next step, 1 µL of the cDNA template was mixed with other reagents including PCR buffer, dNTPs, Taq DNA polymerase, forward and reverse primers (CD34 and CD73, Table I), MgCl_2_ and H_2_O. Then, the microtubes containing the above mixture (20 µL) were transferred into Thermocycler (Eppendorf Mastercycler Gradient, Eppendorf, Hamburg, Germany). Thirty amplification cycles were run; denaturation for 30 sec at 95^o^C, annealing for 30 sec at 67.5^o^C (for CD34) and 63.5^o^C (for CD73) and extension for 30 sec at 72^o^C, primary denaturation for 2 min at 95^o^C and final extension for 5 min at 72^o^C. To define the presence of bands, PCR products were tested with the aid of DNA safe stain using gel electrophoresis in 1.5% agarose gel medium and were visualized by UV radiation of a gel documentation system (UVtec, Cambridge, UK).

**Karyotype analysis**

Cells of early passages and after passage 4 were subcultured at a 1:3 dilution. They were exposed to a 4-hr demecolcemid (Sigma, USA). After 24 hr, trypsin-EDTA (Sigma, USA) and hypotonic KCl (Sigma, USA) were added. Then they were fixed in an acid/alcohol solution. For analysis of the metaphase, using a microscope and an oil immersion objective, the number of the chromosome was evaluated and then imaged by a digital camera on a light microscopy.

**Osteogenic and adipogenic induction**

About 1×10^4^ cells of EnMSCs were put in two 35 mm culture dish (Corning, Germany). The first dish contained control medium composed of DMEM-F12, (Bio West, France) supplemented with 10% FBS (BioIdea, Iran), 1% penicillin/streptomycin (BioWest, France) and 1% L-glutamine (BioIdea, Iran). The second dish contained the osteogenic medium of DMEM-F12 supplemented with 10% FBS, 1% penicillin-streptomycin, L-glutamine, 50 μg/ml L-ascorbic acid-2-phosphate (Sigma, USA), 7 M dexamethasone (Sigma, USA), and 10 mM β-glycerophosphate (Sigma, USA). The media were changed every 3 days. After 3 wk, the cells were fixed with 70% ethanol for 15 min and stained for mineralization using 2% Alizarin red (Sigma, USA) and visualized under a light microscope.

For adipogenic induction, EnMSCs were seeded at the density of 1×10^4^ cells in two 35 mm culture dish. The adipogenic medium consisted of DMEM-F12 supplemented with 10% FBS, 1% penicillin-streptomycin, L-glutamine, 60 μM indomethacin (Sigma, USA), 10 μM insulin (Sigma, USA) and 1 μM dexamethasone (Sigma, USA) were added. Induction lasted 3 wk, and the cells were fixed in 4% formalin for 20 min at 4^o^C and presence of oil droplets was confirmed by oil red O staining (Sigma, USA).

**Ethical consideration**

The experimental protocol was approved based on the recommendations of Research Committee of School of Veterinary Medicine, Shiraz University (no. 1211/A/P, date. 191291). 

**Statistical analysis**

The mean and SE of counted cells in growth curve analysis were compared using independent sample t-test with SPSS software (Statistical Package for the Social Sciences, version version 11.5, SPSS Inc, Chicago, USA). Values of p≤0.05 were considered significant.

**Table I:** Sequences of RT-PCR primers used to quantify the expression of endometrial mesenchymal stem stromal cells’ specific marker (CD73) and hematopoietic stem cells’ specific marker (CD34) in sheep

**Primer**	**Primer sequence**	**Amplicon length (bp)**
CD34-F CD34-R	AGCCCTGTGACTTCTTCCA CCTCTGCCTGTTCCTCCT	211
CD73-F CD73-R	CTGATGATGGACGGAAGG GCTGCTGTTGAGAAGAATGG	134

## Results

**Mesenchymal stem cells’ characters of isolated cells from sheep uterus**

**Figure 1:**
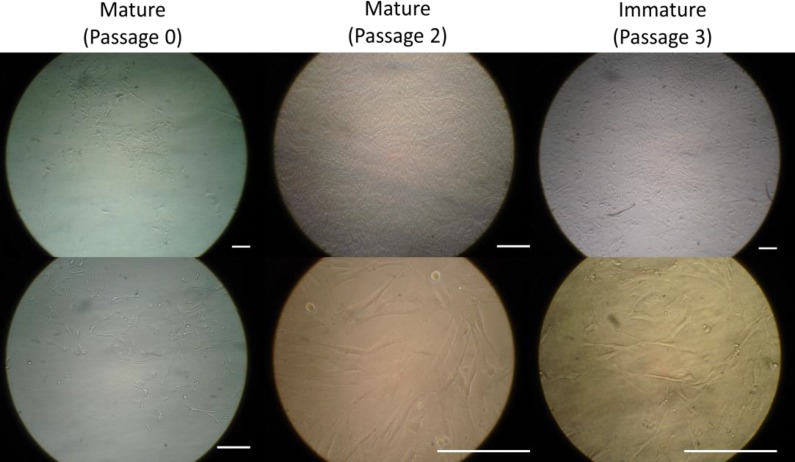
Morphology of ovine endometrial mesenchymal stem stromal cells in mature and immature ewes. Scale bar is 100 μm.

Except for the primary culture (P0) that took 12 days to become confluent, other passages were confluent after three to five days. About 2.2×10^4^ cells per well of the P3 were seeded into 12 well plates to reach the fourth passage (P4) and cell count was undertaken every day until day 8 for both EnMSCs samples of mature and immature ewes. After cell expansion, the EnMSCs of both mature and immature sheep adhered to the flasks were spindle-shaped resembling the morphology of MSCs (Figure 1). 

**Figure 2:**
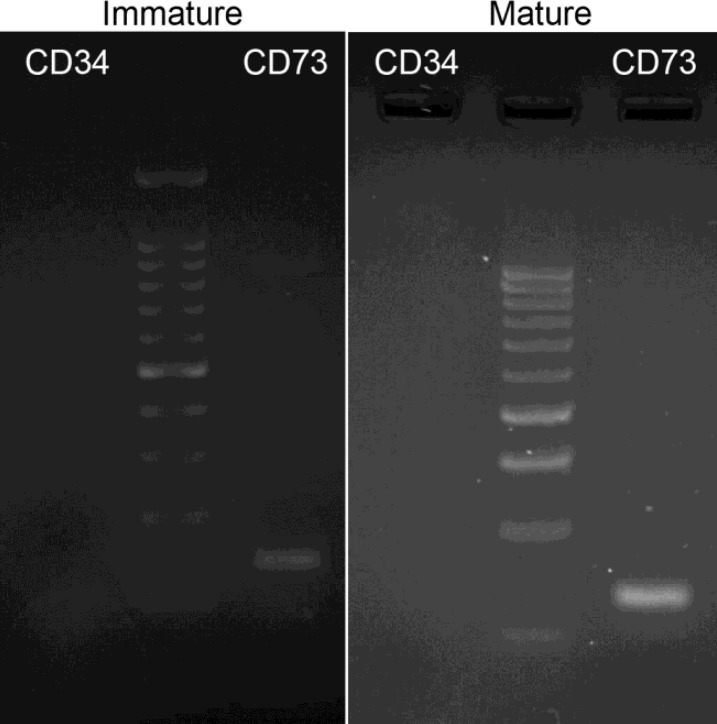
RT-PCR analyses of endometrial mesenchymal stem stromal cells markers. Cells were uniformly positive for CD73 and negative for CD34 in sheep

**Figure 3:**
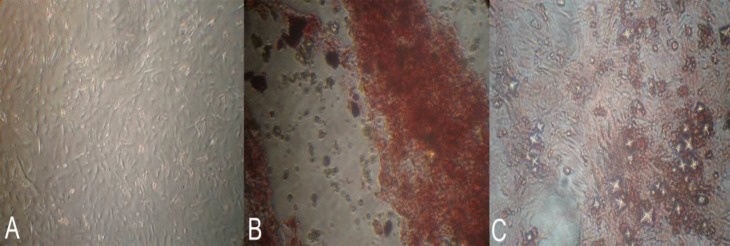
Alizarin red staining of endometrial mesenchymal stem stromal cells of A) control group and B) in osteogenic medium. C) oil red O staining of endometrial mesenchymal stem stromal cells in the adipogenic medium

In addition, RT-PCR analyses of the EnMSCs revealed that these cells were positive for CD73 (MSCs marker) and negative for CD34 (HSCs marker, Figure 2). In addition, data from alizarin red staining of EnMSCs isolated from all ewes in control (Figure 3A) and osteogenic medium revealed the formation of osteoblasts in osteogenic medium (Figure 3B). Following adipogenic induction, the EnMSCs were differentiated toward adipoblasts confirmed by positive oil red O staining (Figure 3C).

**Karyotyping findings**

**Figure 4:**
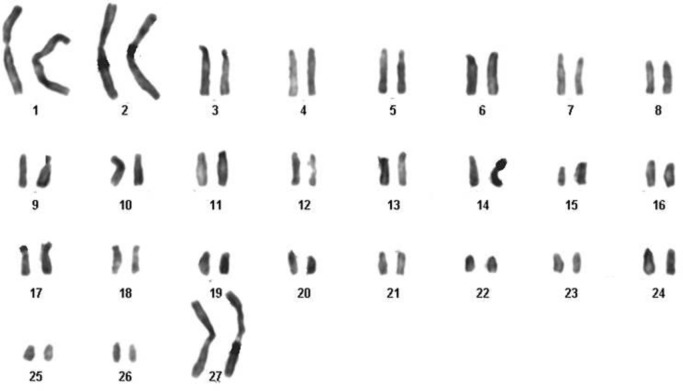
The karyotype of the ovine endometrial mesenchymal stem stromal cells consisted of 27 pairs of chromosomes. The sex chromosome type was XX (♀).

The chromosome number of EnMSCs isolated from sheep in both early passages and after passage 4 was 2n=54, containing 52 autosomal and 2 sex chromosomes (Figure 4).

**Maturity decreases the proliferation of EnMSCs**

Based on the finding of the cell counts, the growth rate of EnMSCs of immature ewes was significantly higher than the value for mature sheep as PDT of EnMSCs of immature ewes was about 21 hr whereas this time period was two times higher (45 hr) in mature sheep. Therefore, immature young animals showed a higher proliferation rate of EnMSCs in comparison to mature sheep. 

**Increase of the seeded cells increases the proliferation of EnMSCs**

**Figure 5:**
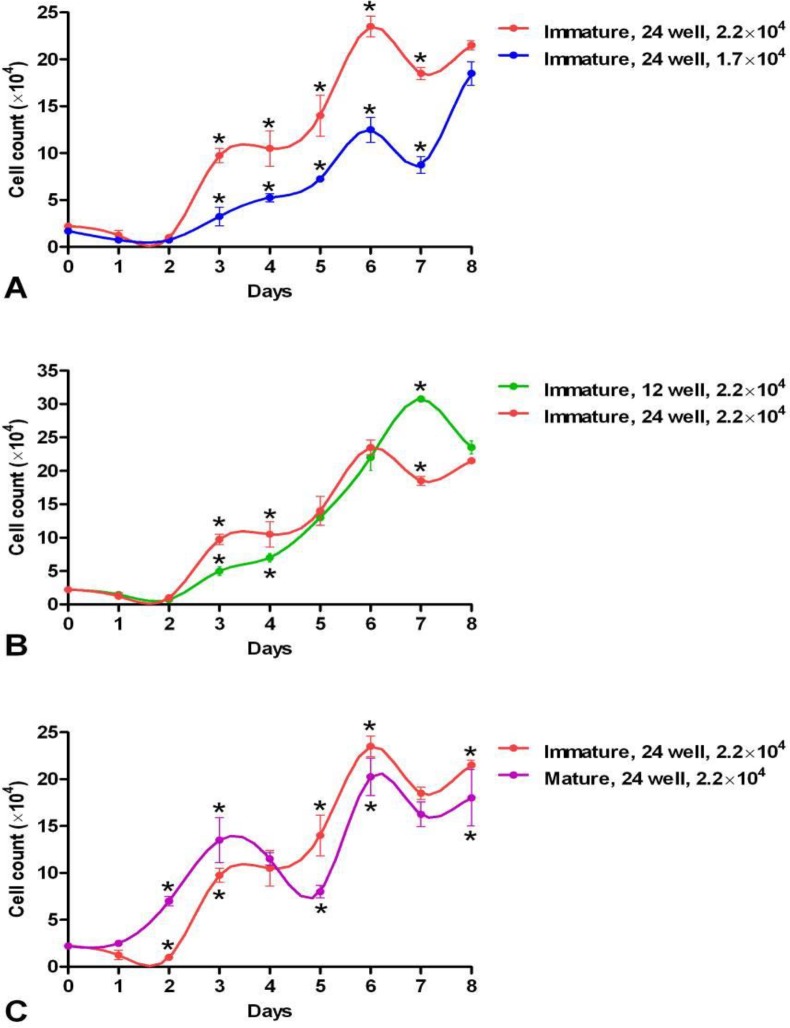
Comparisons of the growth curves of endometrial mesenchymal stem stromal cells A) in immature ewes seeding different cell numbers (2.2×10^4^ and 1.7×10^4^) at the starting point in 24-well culture plates; B) in immature ewes seeding 2.2×10^4^ cells at the starting point in 12- and 24-well culture plates; and C) between mature and immature ewes when seeding 2.2×10^4^ cells at the starting point in 24-well culture plates. * Stars show significant differences between groups in same days (p<0.05

In immature ewes, it was shown that when 1.7×10^4^ cells were seeded at the starting point in a 24-well culture plate, the PDT changed to 23.7 h and the cell count was less than 20×10^4^. When comparing seeding of different cell numbers (1.7×10^4^ and 2.2×10^4^ cells) at the starting point in 24-well culture plates, the cell seeding of 2.2×10^4^ at the starting point yielded better results (Figure 5A).

**Decrease of culturing area of the seeded cells increases the proliferation of EnMSCs**

In immature ovine, when seeding 2.2×10^4^ cells at the starting point in a 12-well culture plate, the PDT reaches 22.4 h and the cell count increases after 8 days to more than 30×10^4^ cells. In these animals in a 24-well culture plate, the PDT was 21.1 h and the cell count reached to less than 25×10^4^ cells (Figure 5B). When comparing seeding of 2.2×10^4^ cells at the starting point in 12- and 24-well culture plates, seeding of cells in a 12-well yielded better results (Figure 5B).

**Sheep maturity decreases the proliferation of EnMSCs**

In mature sheep when using 2.2×10^4^ cells at the starting point in a 24-well culture plate, the PDT changed to 45.2 h and the cell count to less than 20×10^4^ cells. Finally, the comparison between mature and immature sheep regarding seeding of 2.2×10^4^ cells at the starting point in a 24-well culture plate showed that EnMSCs of immature ewes yielded a significant better cell count. Although, EnMSCs from mature ewes displayed better proliferation rates during the first days of the proliferation study (Figure 5C).

## Discussion

In the present study, the EnMSCs were isolated from the uterus of ewes displayed spindle shape and exhibited typical fibroblast-like morphology in culture media. After cell expansion, the most important qualitative parameter is morphology that can be evaluated by light microscopy in a regular pattern. MSCs are defined as plastic adherent cells while EnMSCs having similar phenotype and characters of bone marrow-derived MSCs (BM-MSCs) or adipose tissue-derived MSCs (AT-MSCs) (25, 26). The morphology, cellular phenotype and growth characteristics of the EnMSCs were shown to be very identical to features of the MSCs (27). Plastic adherent property identical to the MSCs seen in other adult tissues was also observed in ewe’s EnMSCs. This morphology referred to the mesenchymal property of these cells isolated from ovine endometrium which has been confirmed in other animals’ EnMSCs (26, 28, 29). 

Based on the finding of the growth curve, the growth rate of EnMSCs in immature ewes was higher than the value for mature sheep. Consistent with our finding, proliferative potential of EnMSCs was evaluated by serially passaging individual epithelial and stromal colony forming unit (CFU) until senescence. Large CFU underwent >30 PDT before senescence, showed their high proliferative potential characteristic of EnMSCs (30, 31).

In the current study, PDT of EnMSCs of immature ewes was less than the value of mature sheep. Adult stem cells are responsible for tissue homeostasis, even provision of replacement cells in routine cellular turnover and for the healing of injured tissues (28). As immature sheep is in the developing stage, higher potential of EnMSCs division in their uterus can be an explanation for this event. Furthermore, clonogenicity, the ability of a single cell to form a colony when cultured at very low densities, was previously shown in human endometrium (29). The first evidence for the presence of endometrial epithelial progenitor cells was provided after cell cloning studies of human endometrial cells (29). Existence of EnMSCs niche in the basalis was further supported by the result of clonogenic cells in inactive endometrium (32).

The present study for the first time compared the EnMSCs for growth kinetics and PDT in mature and immature ewes and these data can be supportive for future studies. We realized that two factors influenced the PDT of EnMSCs in both mature and immature ewes including aging (senescence) and the cyclic steroid hormonal levels. Our findings indicated that the growth of EnMSCs in immature ewes was significantly more in comparison to the mature ones. Our findings are consistent with findings of the other sources of stem cells that emphasize the important role of growth kinetic and PDT in the proliferation of stem cells to be used in cell therapy measures. For instance, using the growth curve and the PDT for BM-MSCs and AT-MSCs in rat; it was shown the predominance of bone marrow derived MSCs (33). In addition, based on in vitro growth data and the PDT, the stem cells from third molar tooth were shown to have a low PDT value (34).

A 2n=54 in karyotyping of our samples indicated that the EnMSCs of all sheep were normal and stable diploid as reported before (35). Moreover, our passages of EnMSCs were positive for MSCs marker of CD73 but negative for a hematopoietic marker of CD34. This appearance referred to mesenchymal nature of these cells which were reported by other authors too (11, 36). 

The MSCs are rare undifferentiated cells seen in several adult tissues with high proliferative properties, self-renewal character, differentiation potential, and clonogenicity or CFU activity (37, 38). In the present study, EnMSCs were shown to have potential to differente multilineage such as adipocytes and osteoblasts which is consistent with previous studies that shows this character in EnMSCs (39). The osteogenic potential of EnMSCs revealed alizarin red S stained regions and after culture in adipogenic differentiation medium, the EnMSCs differentiated toward adipoblasts confirmed by positive staining with Oil Red O. These properties likely indicated to regenerative capacity of the endometrium (40).

## Conclusion

In conclusion, the present study showed that sheep EnMSCs were morphologically similar to MSCs while the RT-PCR confirmed their marker and differentiation potentials. The PDT of these cells revealed that immature ewes could yield more cells in a shorter time. So based on normal karyotype of these cells and their differentiation properties, EnMSCs can open a new window in cell and gene therapies and as a readily available source of regenerative medicine. 

Methods for isolating and characterization of properties of high proliferation, self-renewal, and differentiation of EnMSCs from ovine are developed in this study. Determination EnMSCs in ovine endometrium can be a step forward for applying the large animal model to develop preclinical methods and access to novel materials for tissue engineering. In addition, accessing to this ability for culturing cells open new windows in assisted reproductive technology for research on co-culturing of the ovine embryo with EnMSCs

## Note

***This article extracted from*** ***D.V.M.*** ***Thesis. (Farnaz Ghobadi)***
